# Integrated transcriptomics and metabolomics analyses reveal key pathway responses during the grain-filling stage in maize under waterlogging stress

**DOI:** 10.3389/fpls.2025.1698890

**Published:** 2026-01-19

**Authors:** Bujin Zhou, Shaoli Wei, Jiaming Qin, Jiaxing Zheng, Bingwei Wang, Xiu Zhong, Anxia Huang, Jingdan He, Chengqiao Shi

**Affiliations:** Maize Research Institute of Guangxi Academy of Agricultural Science, Nanning, Guangxi, China

**Keywords:** maize, waterlogging, transcriptomic, metabolomic, grain-filling stage

## Abstract

Crop tolerance to waterlogging significantly influences survival and productivity under waterlogging conditions. Elucidating the molecular mechanisms underlying waterlogging tolerance could facilitate the development of resilient crop varieties through breeding. This study conducted a comparative analysis of the physiological, transcriptional, and metabolic responses of a waterlogging-tolerant maize genotype Guidan162 (GD) and a waterlogging-sensitive genotype Zhaofeng 588 (ZF) during the grain filling stage. Phenotypic and physiological characteristics indicated that the leaf morphology of maize plants is affected, while the levels of peroxidase (POD) and catalase (CAT) and proline significantly increase under waterlogging stress. Transcriptomic analysis identified 3280 and 2260 differentially expressed genes (DEGs) between normal and waterlogged conditions in GD and ZF, respectively. KEGG enrichment analysis of DEGs revealed that pathways related to plant stress tolerance were enriched, including peroxisome, plant hormone signal transduction, and arginine and proline metabolism. In addition, metabolomic profiling revealed 359 and 209 differentially abundant metabolites (DAMs) in GD and ZF under waterlogging stress. Many of these DAMs participate in arginine and proline metabolism, plant signal transduction, and glutathione metabolism. Integrated transcriptomic and metabolomic analyses highlighted significant enrichment in abscisic acid (ABA) signaling, glutathione metabolism, and proline biosynthesis pathways. Several key candidate genes-including Arginase, PIP, P4H, PYR/PYL, PP2C, SnRK2, ABF, IDH, GPX, GGCT, OXP, and GCL were implicated in conferring waterlogging tolerance. These findings provide new insights into the complex molecular mechanisms of waterlogging tolerance in maize.

## 1 Introduction

In recent years, accompanied by global climate anomalies, the number of extreme weather events with uneven rainfall distributions has increased annually, leading to the frequent occurrence of torrential rains and waterlogging disasters in localized areas (Pedersen et al., 2017). As a significant abiotic stress factor, waterlogging not only affects crop growth and development but also reduces yield, sometimes even causing complete crop failure (Voesenek et al., 2006; Wu and Yang, 2016). However, different genotypes present substantial genetic differences in their responses to waterlogging stress. Analyzing the molecular and metabolic regulatory mechanisms in waterlogging-tolerance varieties is crucial for the breeding of new stress-tolerant cultivars.

To understand plant waterlogging tolerance mechanisms, numerous studies have been conducted on the morphological, molecular, and physiological adaptations that occur under waterlogging stress in various plants, including wheat (Cid et al., 2024), barley (De Castro et al., 2022), soybean (Sathi et al., 2022), cotton (Owusu et al., 2023), sorghum (Zhang et al., 2023), and maize (Huang et al., 2022). During long-term evolution, plants have developed multiple strategies to cope with excess water through morphological modifications, gene expression regulation, and metabolic adjustments. Under waterlogging stress, the extremely low diffusion rate of oxygen in water leads to hypoxic conditions in plants. Morphological adaptations such as adventitious root formation, aerenchyma development, and accelerated stem internode elongation help plants survive in hypoxic environments (Oladosu et al., 2020). In addition, oxygen deficiency under waterlogging stress induces the accumulation of intracellular reactive oxygen species (ROS) (Ashraf, 2009; Pan et al., 2021), which can damage the cellular membrane and organelles (Sharma et al., 2012; Baxter et al., 2014). Previous studies have shown that waterlogging-tolerance crops exhibit greater antioxidant enzyme activities and accumulate more low-molecular-weight antioxidants, including glutathione peroxidase (GPX), glutathione, ascorbic acid, and flavonoids, under waterlogging stress (Hasanuzzaman et al., 2020; Chen et al., 2021). In barley leaves, both tolerant and sensitive genotypes presented increased Superoxide Dismutase (SOD), peroxidase (POD) and catalase (CAT) activities under waterlogging stress, but these enzyme activities were significantly greater in tolerant genotypes (Luan et al., 2018). Additionally, abscisic acid (ABA), known as a “stress hormone,” accumulates under various abiotic stresses and mediates plant cross adaptation (Bharath et al., 2021). Studies in cucumber and soybean revealed significantly reduced ABA levels in the roots of waterlogging-tolerance varieties, whereas only minor changes were observed in sensitive varieties (Xu et al., 2014; Kim et al., 2015a). Different crops employ distinct waterlogging tolerance mechanisms, necessitating further elucidation of these protective mechanisms to better understand plant adaptation strategies under waterlogged conditions.

The rapid development of high-throughput omics technologies has enabled comprehensive insights into gene expression and metabolic changes in plants under abiotic stress. Numerous studies have reported transcriptomic analyses of various plants under waterlogging stress, with a primary focus on identifying the dynamic changes in gene expression patterns that occur under such conditions (Zhang et al., 2015; Zhao et al., 2018; Cao et al., 2022; Huang et al., 2023). Metabolomics analysis can complement these findings by revealing physiological states and regulatory networks at the metabolite level (Coutinho et al., 2018). Researchers have employed metabolomics analysis to elucidate complex mechanisms involving cellular metabolic pathways under waterlogging stress, with many metabolites identified as crucial players in waterlogging tolerance (Cui et al., 2019; Cisse et al., 2024; Guo et al., 2024). Metabolites serve as superior targets for enhancing stress tolerance, as they represent the terminal products of gene interactions and directly participate in systemic stress responses (Nakamura et al., 2012). Consequently, the integration of transcriptomics and metabolomics analysis constitutes a powerful systems biology approach for deciphering plant stress response mechanisms.

Maize (*Zea mays* L.), a vital crop globally, plays significant roles in food production, animal feed, and industrial applications. While substantial water is needed for maize growth, excessive moisture severely impacts its development, leading to reduced yields or even crop failure (Tian et al., 2020). With the increasing frequency of extreme climate events, maize production faces increasing environmental challenges, particularly related to waterlogging. Numerous studies have investigated maize waterlogging tolerance (Yu et al., 2015; Ren et al., 2016; Tian et al., 2019). In the V3 and V6 stages of maize, waterlogging disrupts carbon metabolism, alters endogenous hormone levels, accelerates leaf senescence, and ultimately reduces photosynthetic capacity and grain yield (Ren et al., 2016). During seedling-stage waterlogging, signal transduction, along with carbon and amino acid metabolism, plays crucial roles in tolerance mechanisms (Zou et al., 2010). However, to date, research has focused predominantly on seedling-stage responses, with limited investigations of the critical grain-filling stage.

We aimed to explore the changes that occur in maize under waterlogging stress during the grain-filling stage and identify the important pathways for maize adaptation and tolerance to waterlogging stress by examining waterlogging responses during maize grain filling and identifying key pathways involved in waterlogging adaptation. Two maize genotypes, Guidan162 (GD, tolerant) and Zhaofeng588 (ZF, sensitive), were subjected to waterlogging stress and normal irrigation, followed by transcriptomic and metabolomic analysis of ear leaves. RNA sequencing revealed extensive differential gene expression in both genotypes under stress. Complementary LC-MS-based metabolomic profiling revealed metabolic differences between genotypes. Through integrated analysis of transcriptomic and metabolomic data, key metabolic pathways associated with waterlogging tolerance during maize grain filling, along with pivotal candidate genes and metabolites, were identified. These findings provide a scientific basis for the molecular breeding of waterlogging-tolerance maize varieties. This systematic research offers novel insights into the molecular mechanisms underlying waterlogging stress during the grain-filling stage, with potential for applications related to the genetic improvement of maize.

## 2 Materials and methods

### 2.1 Plant materials

In this study, two maize genotypes whose waterlogging tolerances are known to differ were used as experimental materials. GD and ZF are excellent cultivars that were bred by our group, and their characteristics are stable and consistent. GD has good waterlogging tolerance (Wang et al., 2023), but ZF is more sensitive to waterlogging.

### 2.2 Waterlogging stress treatment and sampling

A relatively flat field was selected, and two 5 m × 5 m plots were designated. One plot served as the control. The other plot was excavated to a depth of 60 cm, reinforced with steel frames at the bottom and sides, and lined with a 5 m × 5 m × 1.7 m waterproof cloth. Then the soil was backfilled to the same level as the control plot. For the waterlogging tolerance experiment, the waterproof cloth is pulled up from the edge and fixed on the support frame, and injected water into the waterlogging pool.

The experiment was conducted at the Guangxi Academy of Agricultural Sciences in Nanning, China. The maize seeds were soaked in distilled water overnight, disinfected with 0.1% sodium hypochlorite solution twice for 10 minutes each, and finally washed three times with distilled water. Uniform maize seeds were selected and sown in seedling trays filled with nutrient soil. The seedlings were raised in a controlled environment (greenhouse) with natural light and temperature (28 °C/day and 25 °C/night) and a relative humidity ranging from 70% to 85%. When the maize seedlings grew to the 3-leaf stage, uniformly sized maize seedlings were selected and transplanted into the control pool and the waterlogging-tolerance pool at a density of 54,000 plants/ha; the row spacing was 70 cm, and the plant spacing was 26 cm. The control pool and the waterlogging-tolerance pool were subsequently managed normally according to the maize field. Twelve days after pollination (during the grain-filling stage), water was added to the waterlogging-tolerance pool to a depth of 80 cm. Twelve hours after water injection, the ear leaves of GD and ZF in the control pool and the waterlogging-tolerance pool were collected, frozen in liquid nitrogen and stored at -80 °C for physiological and biochemical analyses and transcriptome and metabolome analyses. Maize plants were subjected to waterlogging treatment for 7 days, with the water depth maintained at 80 cm. After 7 days, the water was drained from the waterlogging pool, and plant morphology was observed. At the same time, the plant continues to grow under natural conditions until fully mature, and the weight of each ear is measured.

### 2.3 RNA extraction, cDNA library construction, and deep sequencing

The total RNA of the leaves was extracted using the Plant RNA Purification Reagent (Invitrogen, Carlsbad, CA, USA) according to the manufacturer’s protocol. During RNA extraction, the gDNA was degraded using DNase I (TAKARA, Beijing, China). The RNA concentration was verified with a NanoDrop2000 spectrophotometer (Thermo Fisher Scientific, Waltham, MA, USA), and the RNA integrity was assessed via agarose gel electrophoresis with an Agilent 2100 Bioanalyzer (Agilent, Palo Alto, California, USA). The RNA samples were evenly divided into two groups for construction of a transcriptome library and RT-qPCR validation.

The RNA-seq library was prepared using the BGISEQ-500 transcriptome library workflow according to the manufacturer’s protocol (BGI, Shenzhen, China). The mRNA was isolated using oligo (dT)-attached magnetic beads and fragmented in fragmentation buffer. The first-strand cDNA was subsequently synthesized with random hexamer priming for reverse transcription, followed by double-strand cDNA synthesis. The synthesized cDNA was subjected to end repair and 3′ adenylation. Adaptors were ligated to the ends of the 3′ adenylation cDNA. The target cDNA fragments were PCR-amplified over 15 cycles using DNA polymerase. Finally, each paired-end library was used for sequencing on a BGISEQ-500 platform, with 2 × 100 bp paired-end reads; original raw data (FASTQ format) were generated. The data presented in the study are deposited in the NCBI Sequence Read Archive (SRA) database, accession number PRJNA1289215.

### 2.4 RNA-seq analysis

The reference genome (Zm-B73-REFERENCE-NAM-5.0.fa.gz) and genome structure annotation files (Zm-B73-REFERENCE-NAM-5.0_Zm00001eb.1.gff3.gz) were downloaded directly from the Maize GDB (https://staging.maizegdb.org/).

To obtain clean reads, the raw paired-end reads were trimmed with the following parameters in Fastp (Chen et al., 2018): reads with more than 50% low-quality bases (Q ≤ 20), reads containing adaptors, and reads with more than 10% unknown bases. The clean reads were subsequently aligned to the reference genome with the default parameters in HISAT2 software (Kim et al., 2015b), and the mapped reads were subsequently assembled into transcripts using StringTie (Pertea et al., 2015).

The RNA-seq data were analyzed using the MetWare Cloud online platform (www.cloud.metware.cn). Feature counts were used to count the number of reads mapped to each gene (Liao et al., 2014), and gene expression levels are presented here as fragments per kilobase of transcript per million fragments mapped (FPKM). On the basis of the gene expression levels, differential expression analysis of pairs of samples was performed using DESeq2 (Love et al., 2014; Varet et al., 2016). The resulting P values were adjusted using Benjamini and Hochberg’s approach to control the false discovery rate. Genes with adjusted P value<0.05 and |log_2_-fold change| ≥ 1 were considered differentially expressed genes (DEGs). GO and KEGG pathway analyses were carried out using Goatools and KOBAS (Ashburner et al., 2000; Mao et al., 2005). GO terms and KEGG pathways with a Benjamini–Hochberg (BH)-corrected p value ≤ 0.05 were considered enriched.

### 2.5 Metabolomic analysis

The samples for metabolomic analysis were prepared according to the standard method for evaluating metabolites. After the leaf samples were freeze-dried and ground, metabolites from 50 mg leaf samples were extracted with 1200 μl of -20 °C precooled methanol:water (7:3, v/v) internal standard extraction solution. The samples were subsequently vortexed every 30 min and vortexed 6 times before being centrifuged at 12000 rpm (RCF = 13800 g) for 3 min at 4 °C. The supernatant was filtered with a microporous membrane (0.22 μm pore size) and stored in an injection bottle for LC–MS/MS analysis. The supernatants of all the samples were mixed in equal volumes to prepare quality control (QC) samples.

The sample extracts were analyzed using a UPLC system (Shim-pack UFLC SHIMADZU CBM30A, Kyoto, Japan) coupled to a tandem mass spectrometry system (Applied Biosystems 4500 Q TRAP, Foster City, CA, USA). The liquid conditions were as follows: UPLC column, Agilent SB-C18 (1.8 µm, 2.1 mm×100 mm); mobile phase, ultrapure water with 0.1% formic acid (solvent A) and acetonitrile with 0.1% formic acid (solvent B); elution gradient, as follows: at 0.00 min, 95% A and 5% B; from 0 to 9 min, 5% B to 95% B; from 9 to 10 min, 95% B; from 10 to 11 min, 95% B to 5% B; and from 11 to 14 min, 5% B to equilibrate the systems. The sample injection volume was fixed at 2 μl, and the flow rate was set to 0.35 ml/min. The column temperature was maintained at 40 °C. The effluent was alternatively connected to an ESI-triple quadrupole-linear ion trap (QTRAP)-MS.

The ESI source operation parameters were as follows: source temperature, 500 °C; ion spray voltage (IS), 5500 V (positive ion mode)/-4500 V (negative ion mode); ion source gas I (GSI), gas II (GSII), and curtain gas (CUR) were set at 50, 60, and 25 psi, respectively; and collision-activated dissociation (CAD) was high. QQQ scans were acquired as MRM experiments with the collision gas (nitrogen) set to medium. Declustering potential (DP) and collision energy (CE) for individual MRM transitions were performed with further DP and CE optimization. A specific set of MRM transitions was monitored for each period according to the metabolites eluted within this period. For two-group analysis, differentially abundant metabolites were identified on the basis of VIP (VIP > 1) and absolute Log_2_FC (|Log_2_FC| ≥ 1.0). VIP values were extracted from the OPLS-DA results, which also included score plots and permutation plots generated using the R package MetaboAnalystR. The data were log transformed (log2) and mean centered before OPLS-DA. To avoid overfitting, a permutation test (200 permutations) was performed. The identified metabolites were annotated using the KEGG Compound database (http://www.kegg.jp/kegg/compound/), and the annotated metabolites were then mapped to the KEGG Pathway database (http://www.kegg.jp/kegg/pathway.html). Pathways with significantly regulated metabolites mapped were then fed into MSEA (metabolite set enrichment analysis), and their significance was determined on the basis of p values obtained hypergeometric test.

### 2.6 Detection of physiological and biochemical indicators

The malondialdehyde (MDA) and proline contents were measured according to Hameed et al., 2012 and Hameed et al., 2014 and Barickman et al., 2019. The activities of antioxidant enzymes, including POD and CAT, were determined by a spectrophotofluorometric method (Shahid et al., 2019). Three biological replicates and three technical replicates of each cultivar treatment and control were analyzed.

### 2.7 Quantitative real-time PCR for RNA-seq validation

Total RNA samples from the GD and ZF treatment and control groups were reverse transcribed into cDNA using HiScript II Q RT SuperMix for qPCR (+gDNA wiper) (Vazyme, Nanjing, China). The relative expression levels of six DEGs from the glutathione, abscisic acid, and proline metabolic pathways were determined by RT-qPCR with the 2^−ΔΔCt^ method. *GAPDH* was used as an internal reference gene. Specific primers were designed with Primer 3 plus (http://www.primer3plus.com/). The primers used for RT-qPCR are listed in **Supplementary Table S7**. RT-qPCR was conducted in a total volume of 15 μl, which consisted of 7.5 μl of SYBR Green PCR master mix (TaKaRa, Dalian, China), 4.9 μl of RNase/DNase-free H_2_O, 0.5 μl of each primer, and 2 μl of cDNA. A Roche 480 Real Time PCR System (Bio-Rad, Hercules, CA, USA) was used to conduct RT-qPCR, and the PCR program was as described previously. All RT-qPCR were performed with triplicate technical replicates.

### 2.8 statistical analysis

Data were subjected to analysis of variance (ANOVA) using SPSS (version 26.0). When the ANOVA indicated a significant effect, means were separated by Tukey’s Honest Significant Difference (HSD) test at a significance level of p < 0.05. All data presented are means ± standard error (SE) of three replicates. All the transcriptome and metabolome visualizations (Venn diagrams, heat maps, scatter plot, circle diagram etc.) were made using an online platform (https://cloud.metware.cn).

## 3 Results

### 3.1 Morphological and physiological responses of GD and ZF under waterlogging stress

**Figure 1:**
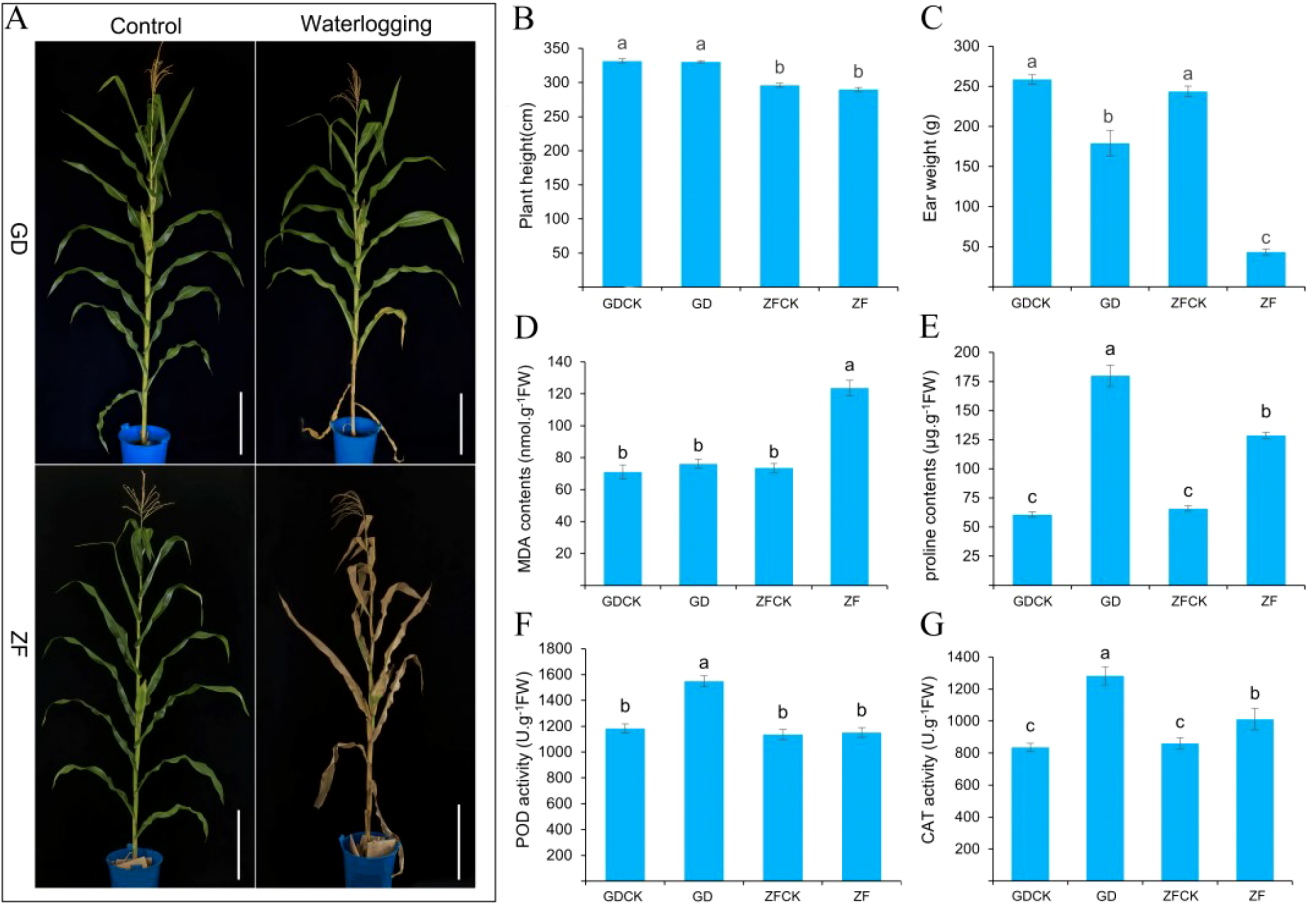
Phenotypic and physiological responses of GD and ZF under waterlogging stress. **(A)** Phenotype of GD and ZF in control and waterlogging-treated groups after 7 days; Bar=50cm. **(B, C)** Plant height and ear weight of GD and ZF in control and waterlogging-treated groups. **(D–G)** Proline and malondialdehyde contents and peroxidase and catalase activity in the leaves of GD and ZF under waterlogging treatment conditions. Significant differences were assessed by ANOVA (p < 0.05).

The plants of GD and ZF were subjected to waterlogging stress 12 days after bagged pollination (grain filling stage). The phenotypes of GD and ZF were investigated after 7 days of waterlogging treatment. GD was more waterlogging tolerant than ZF, with only a few submerged leaves turning yellow. However, the plant leaves have completely withered and died in ZF (**Figure 1A**). No statistically significant alterations in plant height were observed in either GD and ZF before and after the treatment (**Figure 1B**). Although ear weight significantly decreased under waterlogging treatment in both maize cultivars, GD exhibited a significantly higher weight than ZF (**Figure 1C**). In addition, we evaluated physiological and biochemical indicators (MDA, PRO, POD, and CAT) related to waterlogging stress. The proline content of both maize varieties increased under waterlogging treatment, but the GD content was significantly higher than ZF (**Figure 1E**). The waterlogging treatment resulted in increased MDA and proline contents in both maize genotypes. However, the MDA contents of GD were still lower than those of ZF (**Figure 1D**). The activities of POD and CAT increased after waterlogging treatment in the two genotypes. Furthermore, the activities of these enzymes in the waterlogging-sensitive genotype ZF were lower than those in the waterlogging-tolerance genotype GD (**Figures 1F, G**).

### 3.2 DEGs identified in GD and ZF

**Figure 2:**
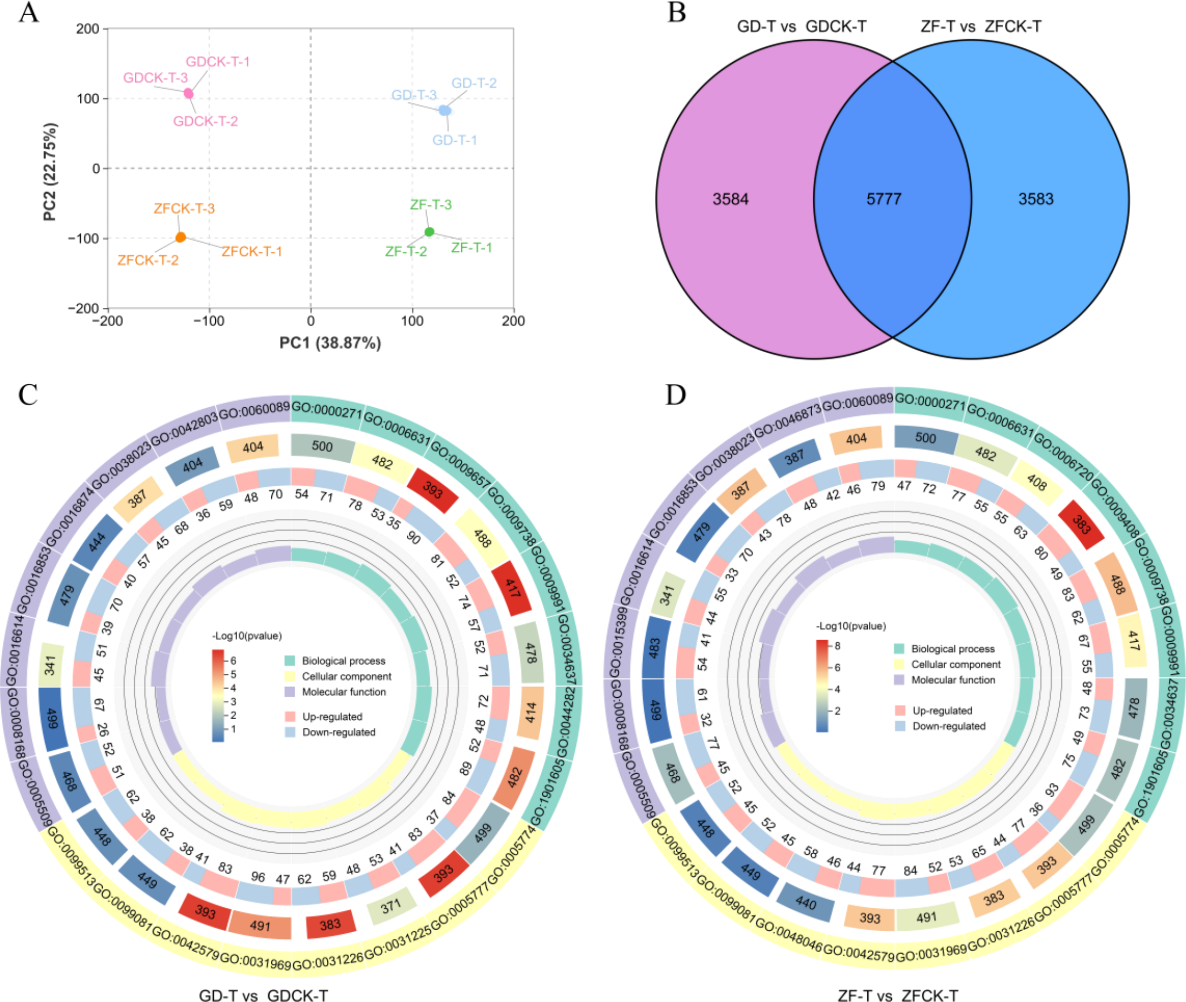
Analysis of transcriptome data. **(A)** Principal component analysis (PCA) of the maize transcriptomes of 12 independent samples from GD and ZF. **(B)** Venn diagram showing the number of total differentially expressed genes (DEGs). **(C, D)** GO Enrichment Circle Diagram of DEGs. From outside to inside, the first circle represents the three major categories of GO entries, with different colors representing different GO classifications; Second circle: The number and q-value of the classification in the background genes. The more genes there are, the longer the bars are, and the more significant the enrichment, the redder it is; Third circle: Bar chart of the proportion of upregulated and downregulated genes, with light red representing the proportion of upregulated genes and light blue representing the proportion of downregulated genes; The specific numerical values are displayed below; Fourth circle: Rich Factor values for each category (the number of foreground genes divided by the number of background genes in that category), with each small grid representing 0.2 in the background auxiliary line.

The transcriptome profiles of the 12 samples were analyzed by principal component analysis (PCA) (**Figure 2A**). The first two principal components explained 38.87% and 22.75% of the variance among samples, respectively. The GD and ZF samples were clearly separated between the waterlogging and well-watered conditions, suggesting significant differences in transcription between these samples.

A total of 9360 DEGs (4233 upregulated and 5127 downregulated, GD-T vs GDCK-T) and 9361 DEGs (4509 upregulated and 4852 downregulated, ZF-T vs ZFCK-T) were identified in GD and ZF, respectively (**Supplementary Table S1**). The number of DEGs is basically the same between GD and ZF (**Figure 2B**). Among these DEGs, 5777 common DEGs were detected between GD-T vs GDCK-T and ZF-T vs ZFCK-T (**Figure 2B**). These common DEGs may be valuable potential candidate genes for improving waterlogging tolerance.

Gene Ontology (GO) analysis is widely used in gene functional annotation and enrichment analysis. The DEGs were classified into biological processes, cellular components, and molecular functions. In the biological process of GD-T vs GDCK-T, the differential genes were mainly enriched in the alpha-amino acid metabolic process, abscisic acid-activated signaling pathway and response to extracellular stimulus. In the cellular component, the most enriched DEGs were the integral component of the chloroplast membrane and the peroxisome. The molecular function mainly included molecular transducer activity and signaling receptor activity (**Figure 2C**, **Supplementary Table S2**). The most differentially enriched genes of ZF-T vs ZFCK-T in biological processes were the abscisic acid-activated signaling pathway and fatty acid metabolic process. The DEGs enriched in cellular components were chloroplast membrane, vacuolar membrane and peroxisome. Molecular transducer activity, calcium ion binding and signaling receptor activity were the main types of genes with molecular function enrichment (**Figure 2D**, **Supplementary Table S2**).

In the two genotypes of varieties, many identical GO terms were found, including abscisic acid-activated signaling pathway, chloroplast membrane, peroxisome, molecular transducer activity, and signal receptor activity. The results indicated that these GO terms participate in waterlogging stress responses and tolerance, playing a crucial role in the waterlogging tolerance among different maize varieties.

**Figure 3:**
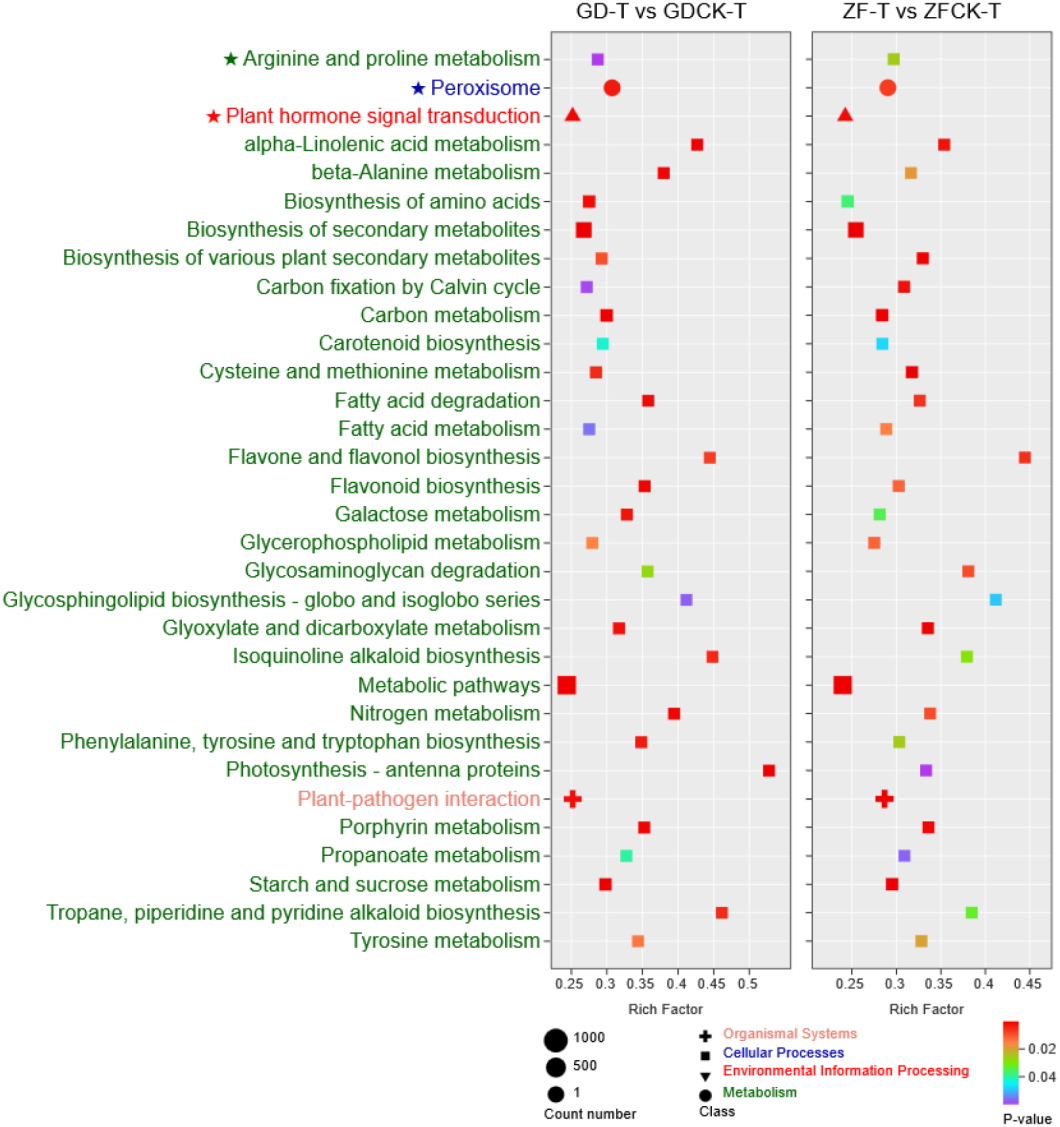
KEGG enrichment analysis of DEGs in GD-T vs GDCK-T and ZF-T vs ZFCK-T. The horizontal axis represents the enrichment factor. A larger factor indicates a greater degree of enrichment. The vertical axis represents the KEGG pathways. The bubble shape denotes the category of the enriched pathway, and the bubble size corresponds to the number of enriched DEGs in the pathway.

Pathway enrichment analysis is an effective method for elucidating the biological functions of DEGs. Therefore, the DEGs enriched in various biological functions were further analyzed by KEGG pathway enrichment. In this study, 43 and 38 pathways were enriched in GD and ZF (p ≤ 0.06), respectively (**Figure 3**, **Supplementary Table S3**). In GD, there are many significantly enriched pathways, including metabolic pathways (ko01100), plant hormone signal transduction (ko04075), plant-pathogen interaction (ko04626), biosynthesis of amino acids (ko01230), arginine and proline metabolism (ko00330) and peroxisome (ko04146) et al. (**Figure 3**). In ZF, metabolic pathways (ko01100), plant hormone signal transduction (ko04075), plant–pathogen interaction (ko04626), MAPK signaling pathway-plant (ko04016), and glycerophospholipid metabolism (ko00564), arginine and proline metabolism (ko00330) and peroxisome (ko04146) were the ten main enriched pathways (**Figure 3**).

The enrichment of KEGG revealed that many pathways related to plant stress tolerance were enriched, such as peroxisome, plant hormone signal transduction, and arginine and proline metabolism. We speculated that these pathways may play vital roles in the response of maize to waterlogging stress conditions.

### 3.3 Differentially abundant metabolites detected in GD and ZF

**Figure 4:**
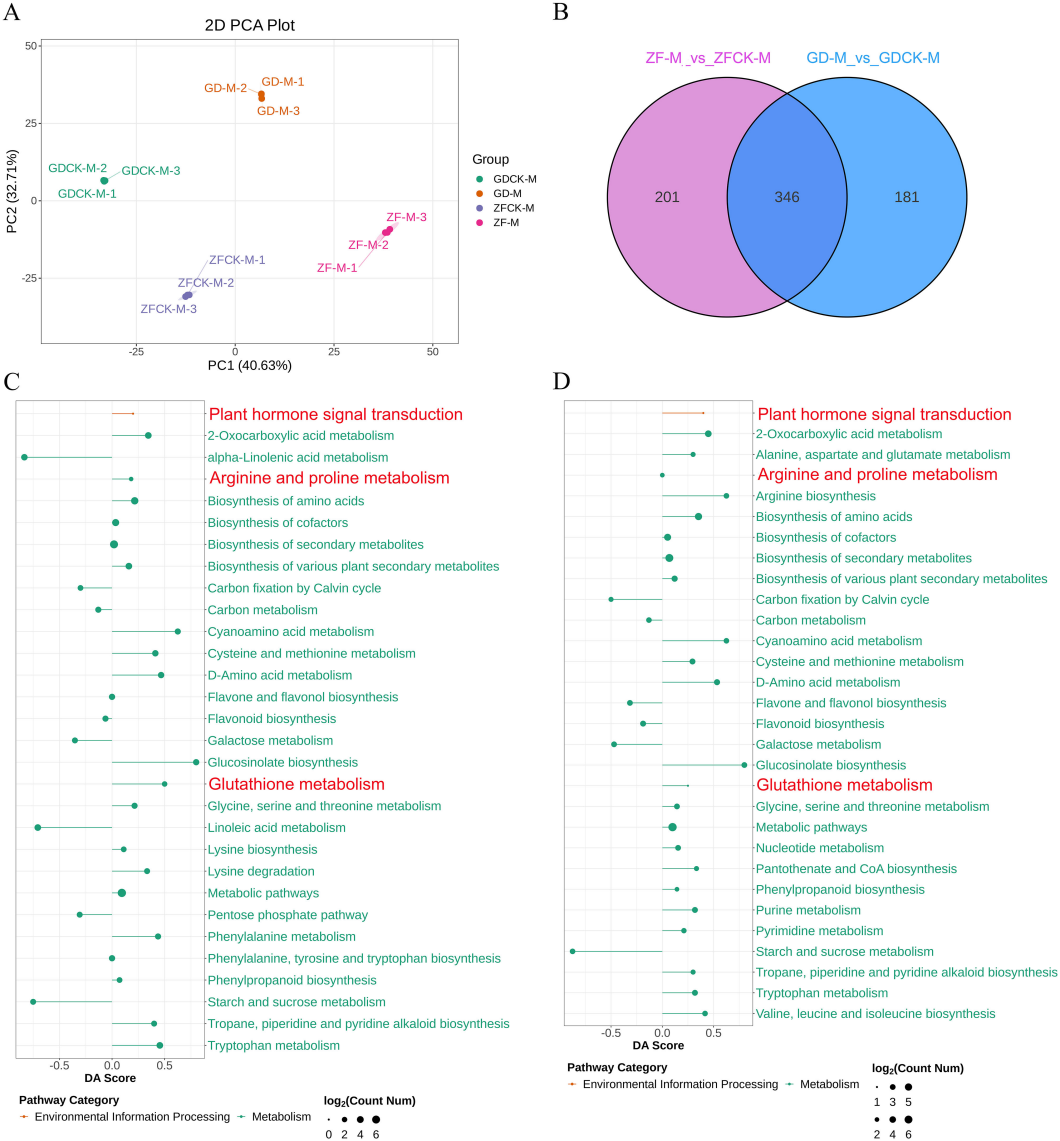
Overview of widely targeted metabolites analysis and KEGG pathway analysis of DAMs. **(A)** Metabolic analysis via principal component analysis (PCA). **(B)** Venn diagram showing the number of total DAMs. **(C, D)** The top 30 pathways of the significance of the GD-M vs GDCK-M and ZF-M vs ZFCK-M DAMs on KEGG. The X-axis represented the DA score, and the Y-axis represented the pathway’s name. The bubble size represents the number of DAMs involved. The bubbles color indicates the enrichment pathway category.

Metabolites can reflect the true physiological state of organisms, and changes in metabolites directly lead to changes in phenotype. In order to explore the response changes of metabolomics characteristics to waterlogging, LC-MS/MS was used to analyze the metabolites of GD and ZF. In total, 1860 metabolites were detected in all leaf samples and were mainly classified into 12 categories: alkaloids (222), amino acids and derivatives (187), flavonoids (401), lignans and coumarins (64), lipids (190), Nucleotides and derivatives (82), organic acids (117), phenolic acids (291), quinones (18), tannins (2), terpenoids (84), others (202) (**Supplementary Table S4**). Principal component analysis (PCA) was used to analyze the dynamic changes in maize under waterlogging stress. The first two principal components could separate 12 samples clearly, accounting for 73.34% of the total variability (**Figure 4A**). The PC1 accounted for 40.63% of the variability, whereas the PC2 accounted for 32.71% of the variability (**Figure 4A**). The results clearly showed that the changes of metabolites in two materials were significantly different, indicating that the response to waterlogging stress was obviously also different.

Metabolites with a variable importance for projection (VIP) value >1 and p<0.05 were considered significant DAMs. Compared with the control group, 527 metabolites were identified as DAMs in GD under waterlogging stress (**Figure 4B**). However, 547 metabolites were identified as DAMs in ZF (**Figure 4B**). Among these metabolites, 346 metabolites were identified in both genotypes. Conversely, 181 metabolites were identified only in GD, and such a number became 201 in ZF (**Figure 4B**).

To further analyze the DAMs related to improving maize waterlogging tolerance, KEGG pathway analyses were carried out for all the DAMs of the two maize genotypes. KEGG enrichment analysis of the DAMs for GD-M vs GDCK-M and ZF-M vs ZFCK-M revealed 81 and 83 metabolic pathways, respectively (**Supplementary Table S5**). KEGG pathway analysis indicated that the DAMs were involved mainly in the biosynthesis of secondary metabolites, the biosynthesis of amino acids and ABC transporters in both GD and ZF under waterlogging stress (**Figure 4C**, **Figure 4D**). Interestingly, arginine and proline metabolism (pme0006, L-Proline), glutathione metabolism (pme1086, Glutathione reduced form; mws4134, Oxiglutatione) and plant hormone signal transduction (Lmtn004049, Abscisic acid) were found to be involved in these KEGG pathways under the waterlogging stress in GD and ZF (**Figure 4C**, **Figure 4D**).

### 3.4 Correlation analysis of DEGs and DAMs during waterlogging

To investigate the relationships between DEGs and DMs in maize during waterlogging treatment, a correlation analysis was performed between DEGs and DAMs in the GDCK vs GD and ZFCK vs ZF comparisons (**Supplementary Figure S1**). An integrated analysis of gene and metabolite responses to waterlogging treatment revealed several commonly enriched pathways, including arginine and proline metabolism, plant hormone signal transduction and glutathione metabolism. These metabolic pathways are closely related to plant stress tolerance.

**Figure 5:**
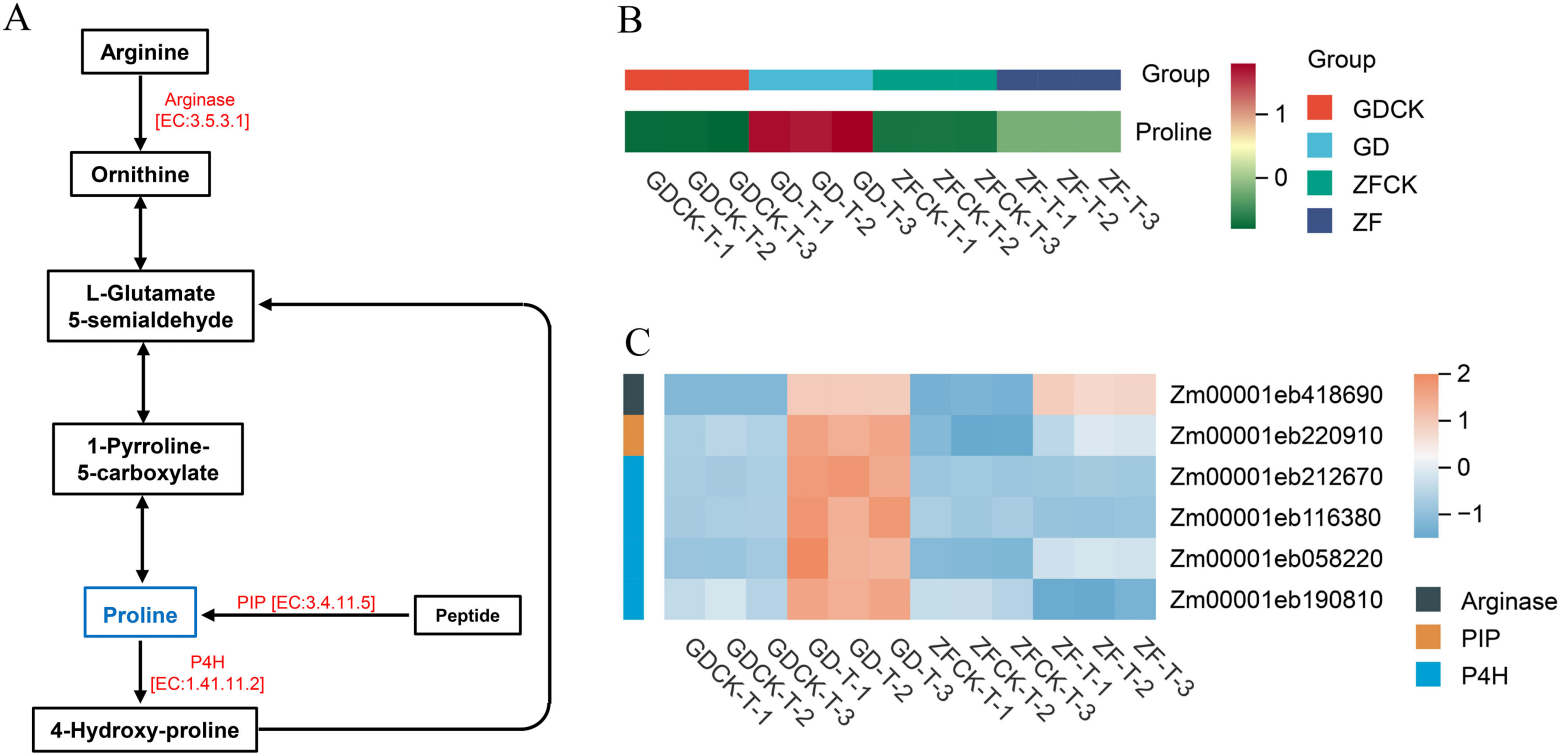
Waterlogging-induced changes in gene expression levels and metabolite contents in the arginine and proline network revealed by the GD vs GDCK and ZF vs ZFCK comparisons. **(A)** The pathways in the center represent the proline regulatory networks. Blue indicates differentially abundant metabolites in response to waterlogging stress. Red indicates proteins expressed in response to waterlogging stress. **(B)** Metabolite abundances under waterlogging stress in the GD vs GDCK and ZF vs ZFCK comparisons. **(C)** Heatmap of DEGs involved in this pathway.

Proline participates in response to stress via its effects on osmotic adjustment. We observed that the proline content in GD and ZF increased under waterlogging stress, mainly through arginine and proline metabolism. However, the proline content in GD was significantly greater than that in ZF (**Figure 5B**, **Supplementary Table S6**). KEGG pathway analysis revealed that arginase (EC:3.5.3.1), proline iminopeptidase (PIP, EC:3.4.11.5) and prolyl 4-hydroxylase (P4H, EC:1.41.11.2), which are involved in the arginine and proline metabolism pathways, were more highly expressed in GD (**Figure 5C**). Analysis of the RNA-seq data revealed that the expression levels of the *Zm00001eb418690* gene, which is functionally annotated as arginase, were increased in both GD and ZF under waterlogging stress, with no significant difference observed between the two genotypes. Although the genes *Zm00001eb220910* and *Zm00001eb058220* were upregulated in both GD and ZF, their expression levels were significantly greater in GD than in ZF. *Zm00001eb212670* and *Zm00001eb116380* were upregulated on GD, whereas ZF exhibited no change in expression. Furthermore, *Zm00001eb190810* was upregulated in GD but significantly downregulated in ZF.

**Figure 6:**
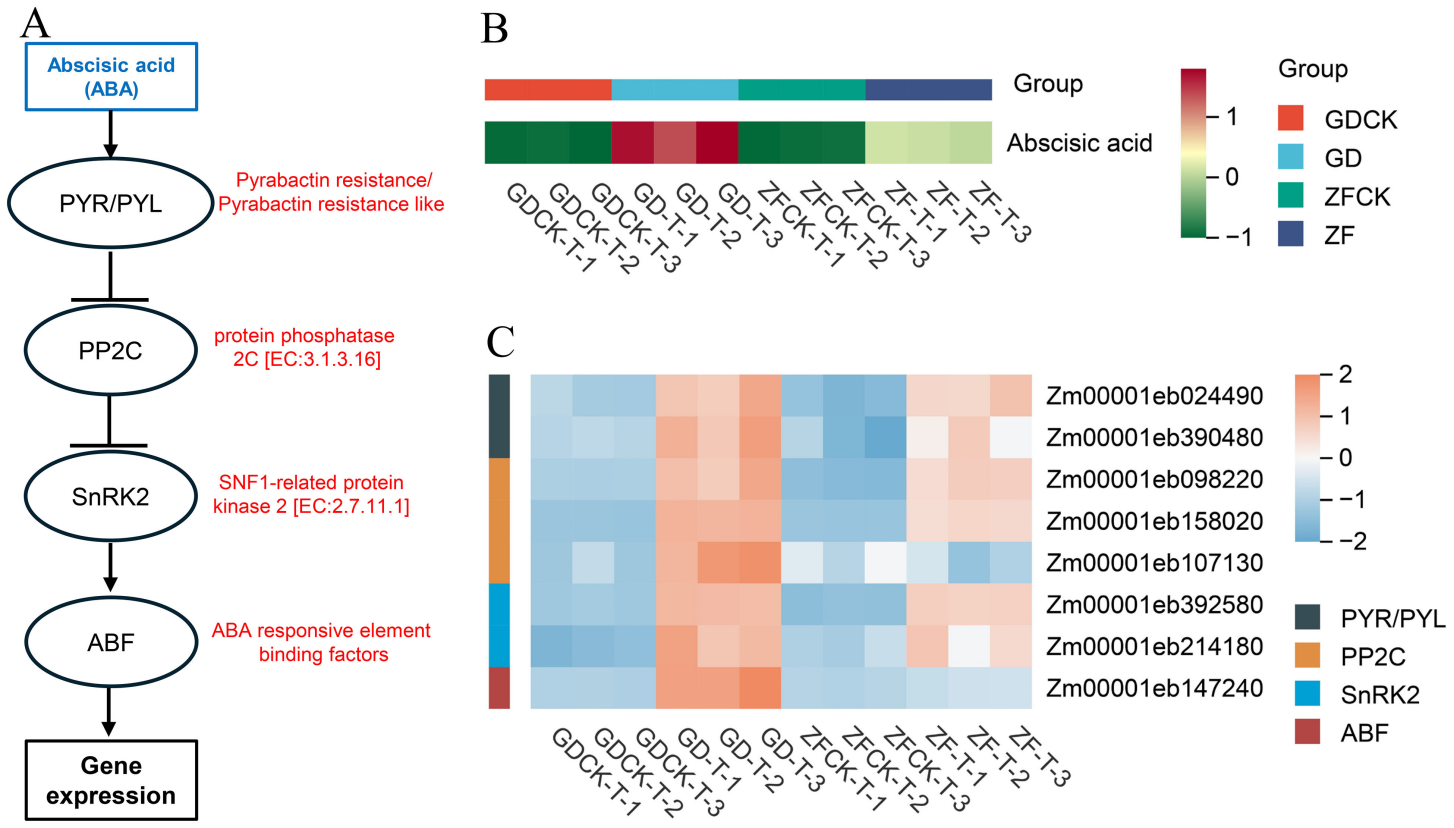
Waterlogging-induced changes in gene expression levels and metabolite contents in the abscisic acid network revealed by the GD vs GDCK and ZF vs ZFCK comparisons. **(A)** The pathways in the center represent the proline regulatory networks. Blue indicates differentially abundant metabolites in response to waterlogging stress. Red indicates proteins expressed in response to waterlogging stress. **(B)** Metabolite abundances under waterlogging stress in the GD-M vs GDCK-M and ZF-M vs ZFCK-M comparisons. **(C)** Heatmap of DEGs involved in this pathway.

Metabolomic analysis revealed that the ABA contents were 12.48- and 5.33-fold greater in the GD-M vs GDCK-M than in the ZF-M vs ZFCK-M, respectively(**Figure 6B**, **Supplementary Table S7**). KEGG pathway analysis revealed that Pyrabactin resistance/Pyrabactin resistance-like (PYR/PYL), protein phosphatase 2C (PP2C, EC:3.3.3.16), SNF1-related protein kinase 2 (SnRK2, EC:2.7.11.1) and ABA responsive element binding factor (ABF) genes were more highly expressed on GD (**Figure 6C**). RNA-seq data analysis revealed that the expression levels of *Zm00001eb024490, Zm00001eb390480, Zm00001eb098220, Zm00001eb158020, Zm00001eb392580* and *Zm00001eb214180* were upregulated in both GD and ZF under waterlogging stress, but their expression levels in GD were greater than those in ZF. In addition, the expression of *Zm00001eb107130* and *Zm00001eb147240* was upregulated on GD under waterlogging stress, whereas their expression levels remained relatively stable on ZF.

**Figure 7:**
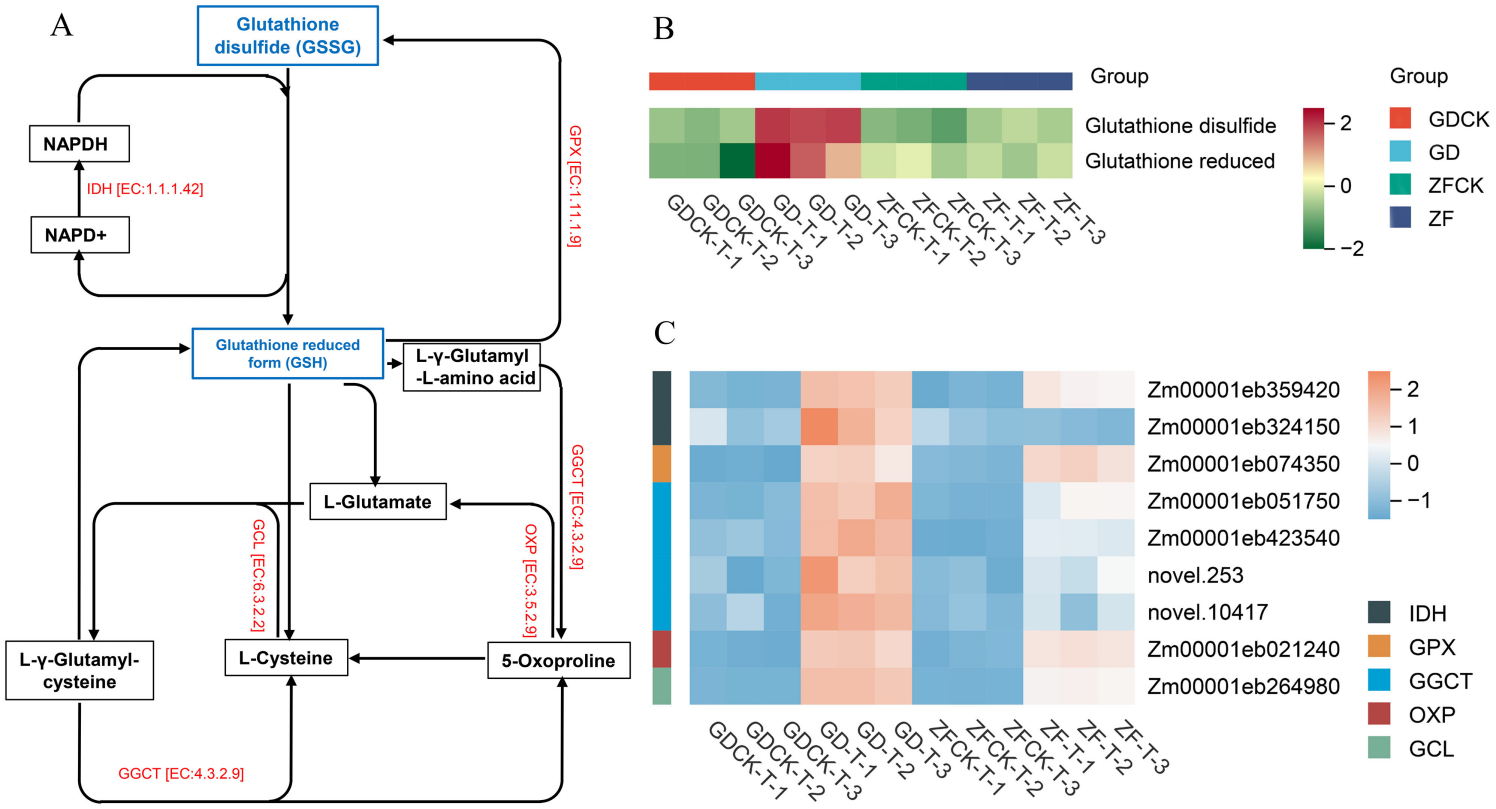
Waterlogging-induced changes in gene expression levels and metabolite contents in the glutathione network revealed by the GD vs GDCK and ZF vs ZFCK comparisons. **(A)** The pathways in the center represent the proline regulatory networks. Blue indicates differentially abundant metabolites in response to waterlogging stress. Red indicates proteins expressed in response to waterlogging stress. **(B)** Metabolite abundances under waterlogging stress in the GD-M vs GDCK-M and ZF-M vs ZFCK-M comparisons. **(C)** Heatmap of DEGs involved in this pathway.

We found that glutathione metabolism was markedly affected, and the detailed network of this pathway was mapped. The glutathione-reduced form (GSH) and glutathione disulfide (GSSG) contents were dramatically increased in GD under waterlogging stress, whereas there were no significant changes in ZF (**Figure 7B**, **Supplementary Table S8**). In addition, key genes in this pathway, including isocitrate dehydrogenase (IDH), glutathione peroxidase (GPX), γ-glutamylcyclotransferase (GGCT), 5-oxoprolinase (OXP), and glutamate-cysteine ligase (GCL), were identified (**Figure 7C**). Interestingly, *Zm00001eb359420*, *Zm00001eb51750, Zm00001eb423540, novel.253* and *novel.10417* and *Zm00001eb264980* were upregulated in both GD and ZF under waterlogging stress. However, the expression levels of these genes were greater in GD than in ZF. The OXP (*Zm00001eb021240*) gene was upregulated in both GD and ZF under waterlogging stress, with no significant difference observed between the two genotypes. The expression level of the *Zm00001eb324150* gene, which is functionally annotated as IDH, was upregulated in GD under waterlogging stress. In contrast, the expression level of the *Zm00001eb324150* gene was downregulated in ZF.

### 3.5 RT-qPCR verification

**Figure 8:**
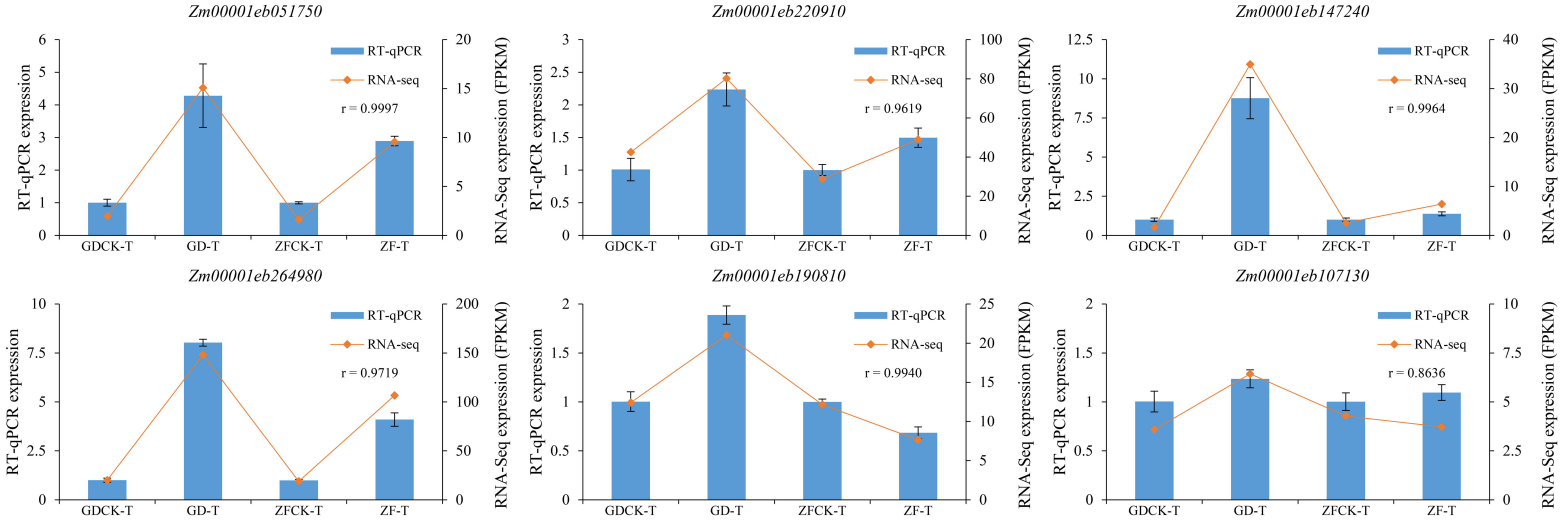
RT-qPCR verification results for six genes. The relative expression levels of the six genes were calculated using the 2^−ΔΔCt^ method. r represents the correlation coefficient between the RT-qPCR data and the RNA-seq data. GDCK-T and ZFCK-T correspond to GD and ZF under normal irrigation conditions, respectively; GD-T and ZF-T correspond to GD and ZF under waterlogging stress, respectively.

To verify the accuracy of our RNA-seq data, six genes associated with the proline metabolism, glutathione metabolism, and abscisic acid metabolism pathways were selected, and their expression patterns were validated by RT–qPCR. The results revealed that the expression patterns of these genes were essentially consistent with the expression patterns observed in the RNA-seq data, indicating that our RNA-seq data were reliable (**Figure 8**, **Supplementary Table S9**).

## 4 Discussion

Water serves as a critical factor regulating the metabolic system of maize throughout its growth and development. However, excessive water can inhibit the normal growth and development of maize and may lead to plant death in severe cases. The grain-filling stage is particularly vital for kernel development in maize, and waterlogging stress during this phase can significantly reduce yield. Through long-term environmental adaptation, plants have evolved various complex mechanisms to cope with waterlogging stress (Kreuzwieser and Rennenberg, 2014; Manghwar et al., 2024). Multi-omics approaches (transcriptomic and metabolomic) have been extensively utilized to identify key metabolic pathways and genes associated with waterlogging tolerance in maize at seedling and V7 stages (Yu et al., 2020; Kaur et al., 2021; Yao, 2021; Li et al., 2025). Nevertheless, the waterlogging tolerance mechanisms during the grain-filling stage remains relatively limited. In the present study, we found that the leaf transcriptomes and metabolomes of GD and ZF differ in many ways under waterlogging stress, namely, in terms of plant hormone balance, the antioxidant system, proline metabolism, etc. These differences are reflected in the trends and degrees of altered gene expression levels and metabolite contents. This general difference between the GD and ZF genotypes is an important reason for the discrepancy in their waterlogging tolerance phenotypes.

### 4.1 Proline involved in waterlogging-stress tolerance

Proline is a component of plant proteins and can also exist in a free form in plant tissues (Hare et al., 1999; Funck et al., 2012). Under stress conditions, the proline content in plants increases significantly (Singh and Singh, 1981; Liu et al., 2023). The accumulated proline not only acts as an osmotic regulator in the plant cytoplasm but also plays important roles in stabilizing macromolecular structures, reducing cellular acidity, detoxifying ammonia, and serving as an energy reservoir to regulate the cellular redox potential (Szabados and Savouré, 2010; Batista Silva et al., 2019). Therefore, the proline content in plants reflects their stress tolerance to some extent, with stress-tolerant varieties typically accumulating relatively high levels of proline (Alvarez et al., 2022). In this study, both metabolomic and physiological-biochemical analyses revealed that proline accumulated under waterlogging stress in GD and ZF (**Figure 1E**). However, compared with ZF, GD exhibited significantly greater proline accumulation (approximately 2.3-3.1 times greater) (**Figure 5B**). Transcriptome analysis further revealed that multiple genes involved in proline metabolism were markedly upregulated in GD under stress conditions (**Figure 5C**). These findings indicate that free proline plays a role in osmotic regulation within maize leaf cells to maintain intracellular homeostasis. The substantial difference in free proline levels between the two varieties may be closely associated with their distinct waterlogging tolerance capacities.

### 4.2 ABA involved in waterlogging-stress tolerance

ABA controls the opening and closing of stomata by regulating the size of guard cells, which helps regulate the water potential in plants (Wu et al., 2022). Moreover, ABA contributes to the formation of root aerenchyma under waterlogging stress (Manghwar et al., 2024). Therefore, ABA is closely related to waterlogging stress (Tong et al., 2021). In the present study, metabolomic analysis revealed that the ABA concentration in the leaves significantly increased after waterlogging stress treatment compared with that of the control in GD (**Figure 6B**). However, the ABA levels in the leaves of ZF plants increased only slightly relative to those in the control plants (**Figure 6B**). At the transcriptional level, a significant number of DEGs in the ABA signal transduction pathway were notably enriched. Furthermore, under waterlogging stress, the expression levels of these genes were upregulated in both genotypes, but their transcriptional abundance in GD was substantially greater than that in ZF (**Figure 6C**). Pretreatment with 10 mM ABA has been reported to have positive effects on the rice net assimilation rate, relative growth rate, and chlorophyll content under submergence (Saha et al., 2021). Similarly, it has been reported that exogenous ABA increases waterlogging tolerance in maize (Hwang and VanToai, 1991; VanToai, 1993), soybean (Komatsu et al., 2013), lettuce (Kato Noguchi, 2000), and Arabidopsis (Ellis et al., 1999). The overexpression of the transcription factor RAP2.6L (AP2/ERF) promoted the upregulation of ABA biosynthetic genes in Arabidopsis, resulting in significant accumulation of ABA. This triggers the activation of the antioxidant defense system and induces stomatal closure, ultimately reducing oxidative damage, delaying senescence, and significantly enhancing waterlogging tolerance (Liu et al., 2012). Overall, ABA accumulation in GD may facilitate stomatal closure, consequently reducing transpiration-mediated water dissipation and ultimately increasing its waterlogging tolerance relative to that of the susceptible cultivar ZF.

### 4.3 ROS involved in waterlogging-stress tolerance

In the context of normal metabolic activities in plants, reactive oxygen species (ROS), such as superoxide radicals (O_2_⁻·), hydroxyl radicals (·OH), and hydrogen peroxide (H_2_O_2_), are generated, and the elimination and production of ROS are constantly balanced to maintain a dynamic equilibrium (Waszczak et al., 2018). Intracellular ROS levels are significantly increased, and their dynamic balance is disrupted under adverse conditions (Yang et al., 2023). These ROS exhibit potent oxidative activity, which can lead to cell membrane lipid peroxidation, oxidative damage to proteins and DNA, and even programmed cell death (PCD) (Tiwari et al., 2002; Baxter et al., 2014; Zhou et al., 2019). MDA, a key indicator of membrane lipid peroxidation, reflects the severity of oxidative stress, and elevated MDA levels directly correlate with increased lipid peroxidation and greater damage to the cell membrane (Cosgrove, 1999; Hameed et al., 2013). In the present study, biochemical analysis revealed that the MDA content increased in both GD and ZF under waterlogging stress. Moreover, the MDA content significantly increased compared with that of the control under waterlogging stress in ZF, but the MDA content slightly increased in GD (**Figure 1D**). These results indicate that the accumulation of ROS is relatively low and that the degree of oxidative damage is relatively small in GD under waterlogging stress, whereas the accumulation of ROS and the degree of oxidative damage are greater in ZF.

To mitigate ROS-induced cellular damage, plants activate their highly efficient ROS scavenging system in response to waterlogging stress. The ROS-scavenging system consists of enzymatic and nonenzymatic antioxidants. Peroxidase (POD) and catalase (CAT) act as key enzymatic antioxidants in this scavenging system, effectively removing free radicals generated during metabolic reactions. Therefore, we analyzed the activities of the POD and CAT enzymes under waterlogging stress. The results indicated that the enzymatic activities of both POD and CAT were significantly increased in GD after waterlogging treatment. However, their activities slightly increased in ZF (**Figures 1F, G**). In addition, GSH plays a crucial role in preventing cellular oxidative damage by balancing the redox state. GSH can not only participate in the clearance of H_2_O_2_ through the AsA–GSH cycle but also directly react with other ROS (May et al., 1998).

In this study, metabolomic analysis revealed that under waterlogging stress, both the GSH and GSSG contents in GD were significantly greater than those in the control, whereas ZF caused no substantial changes in the GSH and GSSG levels before and after treatment (**Figure 7B**). Concurrently, transcriptomic analysis demonstrated that numerous DEGs in the glutathione metabolic pathway were significantly enriched, and these genes in GD were markedly upregulated under waterlogging stress, whereas their expression levels were only slightly increased in ZF (**Figure 7C**). These findings demonstrate that antioxidant enzyme activities are high and that active antioxidant substances are increased in GD, which is beneficial for the ability of plants to scavenge ROS and reduce the degree of cellular oxidative damage caused by waterlogging.

## 5 Conclusions

To explore the changes that occur in maize under waterlogging stress, this study investigated the physiological and biochemical characteristics, key candidate genes and metabolites of maize. The leaves of maize plants presented significant increases in the levels of antioxidant compounds (POD and CAT) and osmoregulatory substances (proline). Additionally, gene/metabolite expression changes in maize were analyzed at multiple levels. Several key candidate genes-including Arginase, PIP, P4H, PYR/PYL, PP2C, SnRK2, ABF, IDH, GPX, GGCT, OXP, and GCL were identified as likely determinants of waterlogging stress tolerance, participating in critical pathways such as arginine and proline metabolism, plant hormone signal transduction (ABA), and glutathione metabolism. Four metabolites (ABA, proline, GSH, and GSSG) were found to be correlated with waterlogging tolerance and were differentially regulated between the two varieties. Integrated analysis of transcriptomic and metabolomic data provides a novel scientific approach to unravel the complex mechanisms underlying maize responses to waterlogging stress. In conclusion, the findings of this study offer valuable insights for addressing waterlogging-related challenges in maize cultivation.

## Data Availability

The datasets presented in this study can be found in online repositories. The names of the repository/repositories and accession number(s) can be found in the article/**Supplementary Material**.
