# Supplementary material for: Integrated transcriptomics and metabolomics analyses reveal key pathway responses during the grain-filling stage in maize under waterlogging stress
Source: Front Plant Sci. 2026 Jan 19;16:1698890. doi: 10.3389/fpls.2025.1698890 (PMC12861905; doi:10.3389/fpls.2025.1698890)
Supplement: Supplementary file 1 [file DataSheet1.zip › Supplementary materials/Figure S1 Correlation clustering heatmap.DOCX]

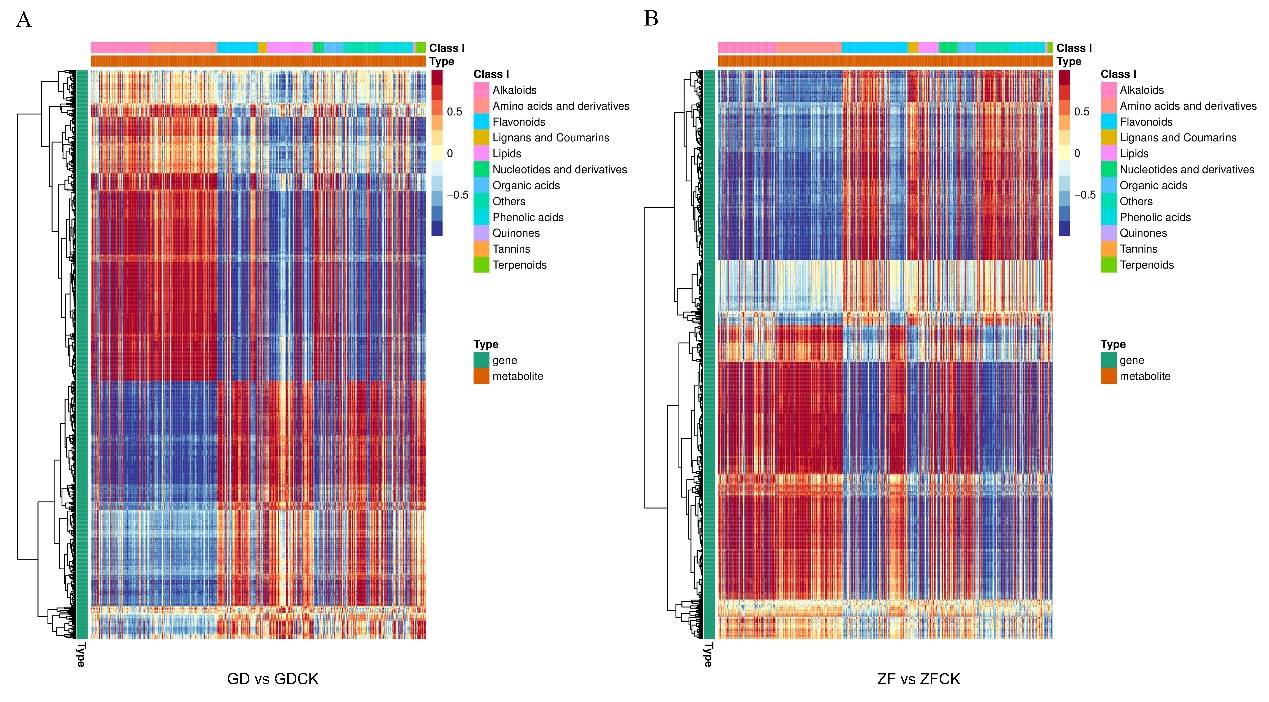


Figure S1 Correlation clustering heatmap

Each row in the figure represents a gene, and each column represents a metabolite. The red color indicates a positive correlation between the gene and metabolite. Blue represents the negative correlation between genes and metabolites. A: GD vs GDCK. B: ZF vs ZFCK.
